# Ethical and Practical Considerations of Artificial Intelligence in Pediatric Medicine: A Systematic Review

**DOI:** 10.7759/cureus.79024

**Published:** 2025-02-14

**Authors:** Hisham Naeem Jamil Abusamra, Salma Hassan M Ali, Wala Ahmed Khidir Elhussien, Alia Mirghani Ahmed Mirghani, Asma Abualgasim Alameen Ahmed, Mohamed Elsayed Abdelrahman Ibrahim

**Affiliations:** 1 Neonatal Intensive Care, Maternity and Children Hospital, Arar, SAU; 2 Obstetrics and Gynecology, Najran Armed Forces Hospital, Najran, SAU; 3 Neonatal Intensive Care, Maternity and Children Hospital, Rafha, SAU; 4 Histopathology, University of Gezira, Wad Madani, SDN; 5 Pediatrics, Alhanakiya Hospital, Madinah, SAU; 6 Internal Medicine, Najran Armed Forces Hospital, Najran, SAU

**Keywords:** artificial intelligence, a systematic review, ethical considerations, machine learning, pediatric medicine

## Abstract

Artificial intelligence (AI) is rapidly transforming various industries, including pediatric medicine, raising both ethical and practical considerations in its implementation. AI is also being utilized more and more in pediatric care across a number of subspecialties. However, a number of major challenges still stand in the way of the practical implementation of AI. The purpose of this systematic review was to evaluate the ethical and practical consideration of AI in the field of pediatric medicine. We searched five different databases (Scopus, Web of Science, PubMed/MEDLINE, EMBASE, and IEEE Xplore) for relevant studies to include in this review. On these databases, we found 524 studies, which were retrieved to EndNote X6.0.1 software, where 207 were removed as duplicates, and the rest were assessed for eligibility with exclusion and inclusion criteria. Twenty studies were found eligible and were included in the study. In pediatric medicine, artificial intelligence (AI) is causing disruption and is now linked to opportunities, problems, and a demand for explainability. AI shouldn't be seen as a replacement for human judgment and experience but rather as a tool to improve and support healthcare decision-making. Therefore, in order to guarantee that research findings are generalizable, future studies should concentrate on gathering complete data.

## Introduction and background

Artificial Intelligence (AI) is increasingly transforming the landscape of modern medicine, offering innovative solutions that enhance diagnosis, treatment, and healthcare delivery [1]. In pediatric medicine, AI holds immense potential to improve patient outcomes through early disease detection, precision medicine, and personalized care [2,3]. AI-driven tools, such as machine learning algorithms, predictive analytics, and robotic-assisted surgeries, are being integrated into various aspects of pediatric healthcare, ranging from neonatal intensive care units (NICUs) to developmental disorder screening and treatment planning. However, despite these advancements, the integration of AI into pediatric medicine raises significant ethical and practical concerns that warrant careful consideration [4].

Patient safety and minimization of harm are among the foremost ethical challenges. There is no question that pediatric patients are uniquely vulnerable because of their developmental stage, inability to provide informed consent, and reliance on guardians in medical decision-making [5]. Thus, the diagnostic and decision support AI-driven systems should be extremely accurate and reliable so that misdiagnoses and inappropriate interventions are avoided. In addition, using AI in pediatric medicine raises questions about data privacy and security since children’s medical records include very sensitive data that need to be very well secured against breach and unauthorized access [6].

Also, there are practical impediments to adopting AI in pediatric medicine beyond ethical considerations [7]. In other words, to implement AI-driven technologies, there is a lot of good financial investment, advanced technical infrastructure, and specialized training for healthcare professionals. Further complications for equitable adoption of AI technologies stem from disparities in access to AI technologies between high- and low-resource settings, which may exacerbate existing healthcare inequalities [8]. Additionally, pediatric datasets do not always generalize as well to adult populations, so developing pediatric-specific models trained on a pediatric population can be necessary, taking into account the distinctive physiological and developmental characteristics of children [9].

Given the rapid advancement of AI in healthcare, a systematic evaluation of both ethical challenges and practical barriers to its integration into pediatric medicine is essential. This review aims to provide a structured synthesis of existing literature, identifying key ethical concerns, implementation challenges, and potential solutions. By offering a comprehensive analysis, this study seeks to inform policymakers, clinicians, and researchers about responsible AI adoption in pediatric healthcare, ensuring a balanced approach that aligns ethical considerations with practical feasibility.

## Review

Methodology

Review Protocol

This study was conducted as per PRISMA (Preferred Reporting Items for Systematic Reviews and Meta-Analyses) 2020 guidelines [10]. Data collection was preceded by establishing a predetermined protocol outlining the goals, inclusion/exclusion criteria, and evaluation techniques. Because of the exploratory nature of the review, the protocol was not published in a public registry. However, any changes from the original plan were openly disclosed.

Eligibility Criteria

After a thorough study by reviewers who assessed whether the papers provided the necessary data for the systematic review, they were selected for inclusion according to the exclusion and inclusion criteria listed in Table 1.

**Table 1:** Eligibility criteria for studies selection.

Inclusion criteria	Exclusion criteria
Studies examining the use of AI in pediatric healthcare.	Studies focusing solely on AI applications in adult populations without pediatric relevance.
Articles discussing ethical concerns such as patient safety, data privacy, informed consent, algorithmic bias, and fairness in AI applications for children.	Articles discussing AI in general healthcare settings without specific reference to pediatric medicine.
Studies addressing practical challenges, including implementation barriers, resource allocation, clinician adoption, and regulatory issues.	Preclinical or purely technical AI studies that do not discuss ethical or practical considerations in pediatric healthcare.
Original research articles, systematic reviews, and qualitative studies published in peer-reviewed journals.	Editorials, commentaries, letters to the editor, conference abstracts, and opinion pieces lacking empirical data.
Studies published in English with accessible full-text availability.	Studies published in languages other than English, unless a high-quality translation was available.

Search Strategy

Scopus, Web of Science, PubMed/MEDLINE, EMBASE, and IEEE Xplore were all thoroughly searched in order to find pertinent studies. A combination of keywords, Boolean operators, and Medical Subject Headings (MeSH) terms were used to create the search strategy. Additional papers relevant to this systematic review were found by manually going through the reference lists of the included papers. To meet the distinctive requirements and indexing words of the corresponding databases, each search string was modified. Table 2 provides the comprehensive search strings for every database.

**Table 2:** Search strategy for each database.

Database	Search string
Scopus	(TITLE-ABS-KEY(("artificial intelligence" OR "machine learning" OR "deep learning" OR "AI") AND ("pediatric" OR "children" OR "infant" OR "adolescent") AND ("ethics" OR "bioethics" OR "privacy" OR "consent" OR "bias" OR "fairness" OR "safety" OR "legal" OR "regulation" OR "equity" OR "practical" OR "implementation")))
Web of Science	(TS=("artificial intelligence" OR "machine learning" OR "deep learning" OR "AI") AND TS=("pediatric" OR "children" OR "infant" OR "adolescent") AND TS=("ethics" OR "bioethics" OR "privacy" OR "consent" OR "bias" OR "fairness" OR "safety" OR "legal" OR "regulation" OR "equity" OR "practical" OR "implementation"))
PubMed/MEDLINE	(("artificial intelligence"[MeSH] OR "machine learning"[MeSH] OR "deep learning"[MeSH] OR "artificial intelligence"[TIAB] OR "machine learning"[TIAB] OR "deep learning"[TIAB] OR "AI"[TIAB]) AND ("pediatrics"[MeSH] OR "child"[MeSH] OR "infant"[MeSH] OR "adolescent"[MeSH] OR "pediatric"[TIAB] OR "children"[TIAB] OR "infant"[TIAB] OR "adolescent"[TIAB]) AND ("ethics"[MeSH] OR "bioethics"[MeSH] OR "privacy"[MeSH] OR "consent"[MeSH] OR "bias"[MeSH] OR "fairness"[MeSH] OR "safety"[MeSH] OR "legal"[MeSH] OR "regulation"[MeSH] OR "equity"[MeSH] OR "practical"[TIAB] OR "implementation"[TIAB]))
EMBASE	('artificial intelligence'/exp OR 'machine learning'/exp OR 'deep learning'/exp OR 'artificial intelligence':ti,ab OR 'machine learning':ti,ab OR 'deep learning':ti,ab OR 'AI':ti,ab) AND ('pediatrics'/exp OR 'child'/exp OR 'infant'/exp OR 'adolescent'/exp OR 'pediatric':ti,ab OR 'children':ti,ab OR 'infant':ti,ab OR 'adolescent':ti,ab) AND ('ethics'/exp OR 'bioethics'/exp OR 'privacy'/exp OR 'consent'/exp OR 'bias'/exp OR 'fairness'/exp OR 'safety'/exp OR 'legal'/exp OR 'regulation'/exp OR 'equity'/exp OR 'practical':ti,ab OR 'implementation':ti,ab)
IEEE Xplore	("artificial intelligence" OR "machine learning" OR "deep learning" OR "AI") AND ("pediatric" OR "children" OR "infant" OR "adolescent") AND ("ethics" OR "bioethics" OR "privacy" OR "consent" OR "bias" OR "fairness" OR "safety" OR "legal" OR "regulation" OR "equity" OR "practical" OR "implementation")

Study Selection

After importing each selected study reference into EndNote (Clarivate 2012, EndNote X (Version 6.0.1), duplicates were removed during the extraction process from various databases. The titles and abstracts were checked for relevancy by two unbiased reviewers from the contributor's list. Both the inclusion and exclusion criteria were used to evaluate studies for which full-text papers were accessible. A third reviewer (tiebreaker) was used to settle disagreements between the first two reviewers.

Data Extraction

Data from included studies was gathered using a standardized Microsoft® Excel Spreadsheet (Microsoft, Inc., Redmond, Wash., USA). Study parameters, including author, year of publication, study design, objectives, outcomes, and key findings, were all included in the extracted data. Data extraction was performed independently by two reviewers, and any discrepancies were resolved through discussion with a third reviewer to ensure accuracy and consistency.

Data Synthesis

Findings from various study designs were integrated using a narrative synthesis approach. Practical and ethical factors were divided into these categories. A meta-analysis was not conducted because of the heterogeneity in study designs and results.

Quality Assessment

The risk of bias assessment was conducted using the Joanna Briggs Institute (JBI) Critical Appraisal Tools. Each of the 20 included studies was evaluated based on eight key criteria: assessing inclusion criteria clarity, detailed study subject and setting descriptions, exposure measurement validity and reliability, use of standard condition assessment criteria, identification and management of confounding factors, outcome measurement validity and reliability, and statistical analysis appropriateness. Each criterion was marked as "Yes" (√) if met, and the percentage of affirmative responses was calculated for each study. Based on the total percentage, studies were categorized as low risk (≥75% "Yes" responses), moderate risk (50-74%), and high risk (<50%).

Results

Search Results

**Figure 1:**
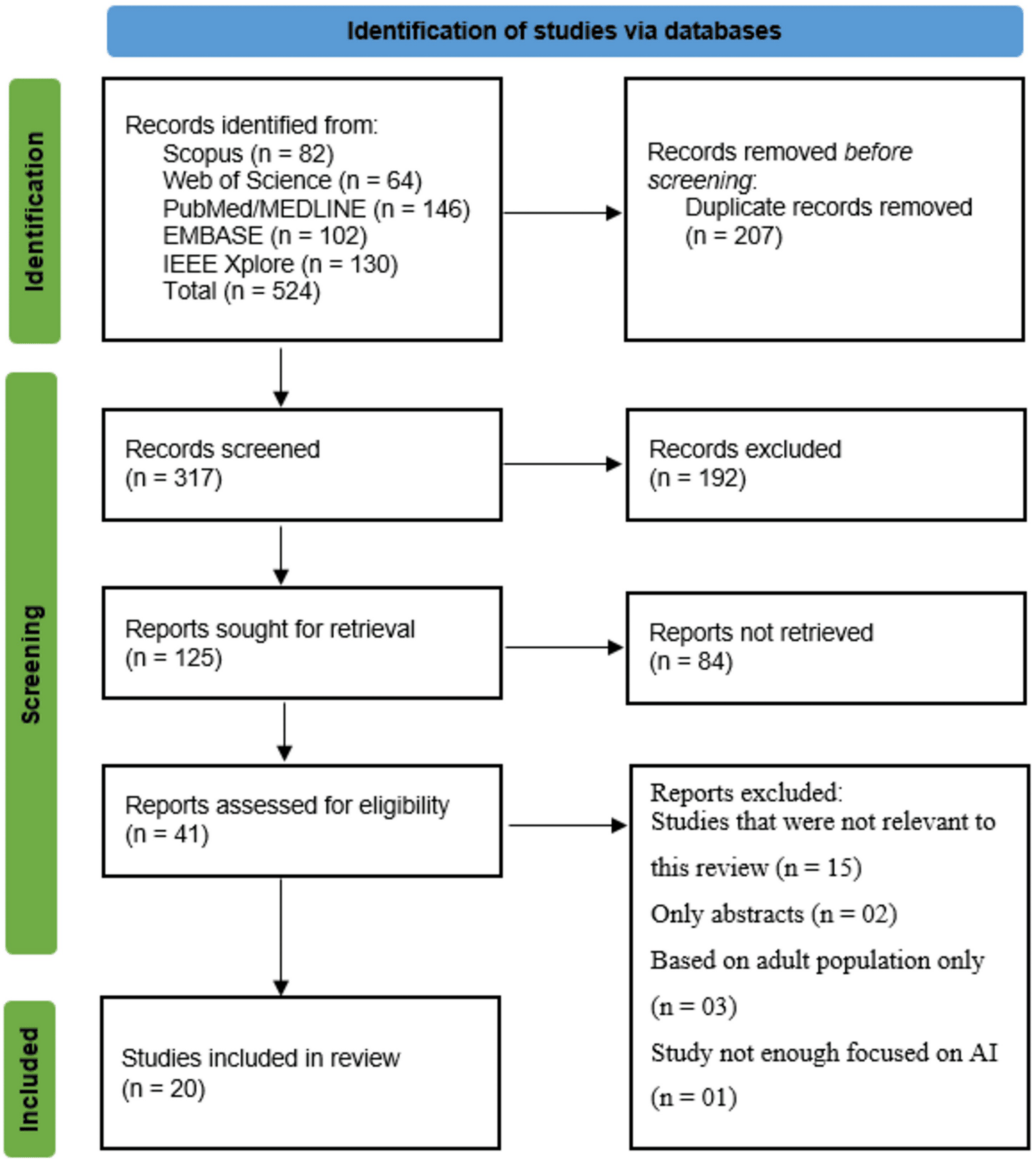
PRISMA flow diagram. PRISMA: Preferred Reporting Items for Systematic Reviews and Meta-Analyses.

A total of 524 records were identified from five databases: Scopus (82), Web of Science (64), PubMed/MEDLINE (146), EMBASE (102), and IEEE Xplore (130). After removing 207 duplicates, 317 records remained for screening. Following title and abstract screening, 192 records were excluded because the studies were not relevant to our systematic review. Full-text retrieval was attempted for 125 reports, but 84 were not retrieved due to full-text open access restrictions. Of the 41 full-text reports assessed for eligibility, 21 were excluded; due to irrelevance (15), abstract-only availability (2), focus on adult populations (3), or insufficient emphasis on AI (1). Ultimately, 20 studies were included in this systematic review (Figure 1).

Characteristics of the Included Studies

The 20 studies included in this systematic review examined various ethical and practical considerations of AI in pediatric medicine. These studies encompassed diverse AI applications, including machine learning (ML) models for disease diagnosis and prediction, AI-assisted clinical decision-making, and the role of AI in improving healthcare workflows. Among them, six studies involved primary data collection and employed ML models such as XGBoost, ANFIS, and logistic regression to assess AI’s effectiveness in diagnosing conditions like child abuse, PTSD, leukemia, PKU, myopia, and thyroid nodules. Five systematic and extensive literature reviews explored AI’s ethical implications, highlighting concerns about data privacy, bias, and the need for regulatory frameworks. Additionally, four qualitative and secondary analyses focused on AI's broader impact, discussing technological challenges, policy considerations, and the need for clinician adoption. The studies varied in their methodologies, including longitudinal analyses, systematic reviews, and primary observational research, with most emphasizing AI's potential to enhance diagnostic accuracy, treatment efficiency, and healthcare decision-making while acknowledging the associated ethical risks and implementation challenges (Table 3).

**Table 3:** Characteristics, outcomes and key findings of included studies. AI: artificial intelligence, ML: machine learning, XGBoost: eXtreme gradient boosting, ANFIS: adaptive neuro-fuzzy inference system, PTSD: post-traumatic stress disorder, PKU: phenylketonuria, EHR: electronic health records, API: application programming interface, PM: precision medicine, CRC: chronic respiratory conditions, RDs: rare diseases, mHealth: mobile health, GDPR: general data protection regulation, HIPAA: health insurance portability and accountability act, CI: confidence interval.

Author and publishing year	Aim	Methodology	Outcomes	Findings
Amrit et al., 2017 [11]	To assess the efficacy of ML and text mining in predicting child abuse cases	Primary data collection from child specialties. Unstructured data is processed in ML algorithms for extraction and classification	The API was found to be the most potential ML model to achieve the predictive classification of child abuse cases	ML can convert clinical unstructured notes and EHR into structured data. ML can characterize cases of abuse based on information extracted from structured data
Ge et al., 2020 [12]	To predict PTSD with diagnostic accuracy in children survivors of an earthquake using machine learning models	XGBoost, an ML model trained on children's clinical data. Children were survivors of disaster	ML achieved 60-80% diagnostic accuracy while predicting PTSD in children suffering from accidental trauma of a natural disaster	ML can detect important factors or variables such as lifestyle deterioration and property loss and generate inferences for the impact of PTSD and its possible consequences in children
Fathi et al., 2020 [13]	To carry out diagnostic analysis of leukemia in children using ML	ANFIS as ML models were used for data inference. Comparative data analysis was performed	An ML-based neuro-fuzzy interface system can best differentiate between paediatric cancer patients and non-cancer patients	NFIS can use children's clinical information to diagnose leukaemia cases and work on the same principles of data extraction, mining, and inference
Zhu et al., 2020 [14]	To demonstrate improvement in the diagnosis of PKU in children using ML models	The logistic regression analysis model with the aim to minimize false-positive rates in the results of the initial PKU test	ML screening models can detect PKU cases with better efficacy at 95% CI	ML is able to extract metabolic data of children and perform data precision to suggest the diagnostic efficacy of PKU cases
Lin et al., 2018 [15]	To predict myopia development in Chinese school-age children group using refraction data in ML	A longitudinal study is collecting children's refraction data over the period 2005-2015. ML algorithms are used to achieve the prediction of myopia development	The results showed a prediction of long-term myopia development in children over the period and with better accuracy	It was found that ML can use refraction values extracted from the clinical records of children in the standard structured form and interpret findings for the development of myopia
Anagnostopoulou et al., 2020 [16]	To review a representative description of the role that artificial intelligence plays nowadays in the assessment of autism	An extensive literature review	The review suggests early and accurate diagnose is the key point for an individualized and successful intervention which aids the academic as well as the personal development of the child	There are some applications of artificial intelligence that are used already or are in a preliminary phase aiming to highlight the use of smart technology in the diagnosing process of autism
Brasil et al., 2019 [17]	To list several examples of how AI has boosted therapeutic development in RDs	An extensive literature review	RDs’ AI-mediated knowledge could significantly boost therapy development	AI boosted therapeutic development in RDs, entailing the identification of disease biomarkers, the increase of patient recruitment, and the discovery of drugs for repurposing. No "one size fits all” AI solution in biomedicine
Huang et al., 2022 [18]	To assess the role of AI in the analysis of pediatric brain tumour imaging and its clinical impact	A systemic review	AI may streamline clinical workflows by improving diagnostic accuracy and automating basic imaging analysis tasks. However, the adoption of AI in clinical practice requires further characterization of validity and utility	Using AI Tumor diagnosis was the most frequently performed task (14, 64%), followed by tumor segmentation (3, 14%) and tumour detection (3, 14%)
Hunt et al., 2020 [19]	To discuss possible entry points for Artificial Intelligence (AI), big data, and mHealth approaches to violence prevention against children, linking these to the World Health Organization's seven INSPIRE strategies	An extensive literature review	The indicators of predictors of violence could be integrated into routine health or other information systems and become the basis of Al algorithms for violence prevention and response systems. However, developing AI and other technological infrastructure will require substantial investment, particularly in low- and middle-income countries	The findings show a clear direction for technology-enabled violence prevention. There is a need to develop reliable and valid population and individual/family-level data on predictors of violence
Kalhori et al., 2021 [20]	To review the application of health information technology, especially artificial intelligence (AI) methods, in treating childhood diseases using precision or personalized medicine (PM)	A systemic review	Though the largest number of clinical articles are devoted to oncology, the analysis showed that genomics was the most PM approach used regarding childhood disease	The number of published papers on AI for PM in childhood diseases increased from 2010 to 2019. The most applied methods were machine learning algorithms
Shah et al., 2023 [21]	To review the applications and limitations of machine learning techniques to empower clinicians to make informed decisions at the bedside	An extensive literature review	It summarizes machine learning and artificial intelligence techniques that are currently in use for clinical data modeling and are relevant to pediatric critical care	Various forms of clinical decision support utilizing machine learning are described. Highlighted the applications and limitations of ml techniques within clinical context, which aid providers in making more informed decisions at the bedside
Clarke et al., 2022 [22]	To discuss the review of future medicine in paediatric	A review methodology	The results suggested that ML can improve in multiple areas, such as improving workload efficiency, diagnostics, precision, precision medicine, and drug development	The findings revealed that AI could facilitate several opportunities in the future and in clinical decision-making to improve pediatric clinical care
Filipow et al., 2022 [23]	To provide a scoping review of ML-based prognosis of chronic respiratory condition in pediatrics	A scoping review (qualitative data analysis)	ML significantly predicted CRC cases in almost all included studies, with results of cases of asthma (80%), cystic fibrosis (12%), and 4% childhood wheezing and 4% bronchitis	The cases of chronic respiratory disease are difficult to manage because of the severity of the symptoms. ML prediction models in all included literature demonstrated the successful diagnosis of cases
Radebe et al., 2021 [24]	To assess ML-based diagnosis of thyroid nodules and clinical decision support	A primary study using a random forest model on biopsy data of children in tertiary care	The model predicted non-benign/malignant cases of thyroid nodules in children. The decrease in false-positive rate and increased accuracy were anticipated to be achieved in the study outcomes	The findings showed that ML could also use clinical cytological data to interpret useful findings, such as from the biopsy results of patients
Shu et al., 2019 [2]	To review the past, present, and future perspectives and outlooks of artificial intelligence in paediatric medicine	An extensive literature review	The results showed that in the future, AI and ML models would have profound implications with the inclusion of Big Data, cloud computing, virtual assistance, clinical decision support, and precision medicine	Big Data and cloud computing technology can handle large datasets, and heterogeneous classification of data would ease with such systems in AI, and therefore precision medicine and clinical decisions would be supported
Aylward et al., 2022 [9]	To provide a brief primer on AI in health care. Introduce AI methods & evaluation metrics	A qualitative analysis	AI approaches promise to reduce barriers to care and maximize the time clinicians are able to spend with their patients	The clinical utility of AI metrics is presented. Future research is still needed to address impediments to widespread clinical adoption
McCartney, 2018 [25]	To provide a discussion paper highlighting the challenges of AI in paediatric medicine	A secondary qualitative analysis was done on the literature	AI faced rigor, authenticity, and challenges in the evaluation of technology, such as how Babylon, a UK app, received from the market	The findings revealed that the Babylon app was not tested for clinical diagnostic accuracy for symptom checking
Vogl M, 2020 [26]	To review the experience of child duplication records in the child welfare sector and problems of AI	A secondary review	The results suggested that data quality issues in child welfare and protection services become barriers to data collection and mining in AI	The data quality issues can cause misinterpretation and misattributions, which may change the inference of clinical findings of children and propose false-positive results
Xiao et al., 2018 [27]	To identify the opportunities and challenges of artificial intelligence	A systematic review (Secondary data collection with thematic analysis)	There are still unnoticeable challenges in the AI associated with a lack of labels and clinical complexity	The findings revealed that clinical complexities are mainly found in the long-term investigation, temporality and irregularity
Davendralingam et al., 2021 [28]	To discuss the challenges of paediatric imaging	A qualitative analysis	The results concluded that data security issues and legal and ethical aspects of clinical challenges	The findings concluded that ethical issues, standardized clinical terms, and problems in their interpretation are causing challenges and opposing the introduction of AI

Out of 20 included studies, 17 (85%) were classified as low risk of bias, demonstrating strong methodological quality with scores ranging from 75% to 100%. Two studies (10%) exhibited moderate risk (50-74%), indicating some methodological limitations, particularly in confounding factor management and statistical analysis [17,26]. Only one study (5%), Anagnostopoulou et al. [16] (2020), was categorized as high risk (37.5%), which indicates significant gaps in study design, inclusion criteria, and confounding factor management. These findings highlight the overall robustness of the included literature while identifying areas requiring methodological improvement in some studies (Table 4).

**Table 4:** Quality assessment of included studies using Joanna Briggs Institute (JBI) Critical Appraisal Tool. Q1. Were the criteria for inclusion in the sample clearly defined? Q2. Were the study subjects and the setting described in detail? Q3. Was the exposure measured validly and reliably? Q4. Were objective, standard criteria used for measurement of the condition? Q5. Were confounding factors identified? Q6. Were strategies to deal with confounding factors stated? Q7. Were the outcomes measured validly and reliably? Q8. Do you know if appropriate statistical analysis was used? (√) - Yes; (-) - No.

Authors	Q1	Q2	Q3	Q4	Q5	Q6	Q7	Q8	% Yes	Risk
Amrit et al., 2017 [11]	√	√	√	√	√	√	√	√	100	Low
Ge et al., 2020 [12]	√	√	√	√	√	√	√	√	100	Low
Fathi et al., 2020 [13]	√	√	√	-	√	√	√	√	87.5	Low
Zhu et al., 2020 [14]	√	√	√	√	√	√	√	√	100	Low
Lin et al., 2018 [15]	√	√	√	√	√	√	√	√	100	Low
Anagnostopoulou et al., 2020 [16]	-	­-	-	√	√	-	√	-	37.5	High
Brasil et al., 2019 [17]	-	-	√	√	√	√	√	-	62.5	Moderate
Huang et al., 2022 [18]	√	√	-	√	√	√	√	-	75	Low
Hunt et al., 2020 [19]	√	√	√	√	√	√	√	-	87.5	Low
Kalhori et al., 2021 [20]	√	√	√	√	√	√	√	-	87.5	Low
Shah et al., 2023 [21]	√	√	√	√	√	√	√	-	87.5	Low
Clarke et al., 2022 [22]	√	-	√	√	√	√	√	-	75	Low
Filipow et al., 2022 [23]	√	√	√	√	√	√	√	-	87.5	Low
Radebe et al., 2021 [24]	√	√	√	√	√	√	√	√	100	Low
Shu et al., 2019 [2]	√	√	√	√	√	√	√	-	87.5	Low
Aylward et al., 2022 [9]	√	√	√	√	√	√	√	-	87.5	Low
McCartney M, 2018 [25]	-	√	√	√	√	√	√	-	75	Low
Vogl M 2020 [26]	-	√	√	√	√	-	-	-	50	Moderate
Xiao et al., 2018 [27]	√	√	√	√	√	√	√	-	87.5	Low
Davendralingam et al., 2021 [28]	√	-	√	√	√	√	√	-	75	Low

Discussion

Artificial intelligence (AI) is increasingly transforming pediatric medicine by improving diagnostic accuracy [29], enhancing clinical decision-making [6], and streamlining healthcare processes [30]. The studies included in this systematic review reveal both the promise and the challenges of AI in pediatric healthcare, highlighting significant ethical and practical considerations. This discussion synthesizes key findings from the 20 selected studies, categorizing insights into diagnostic applications, ethical concerns, implementation barriers, and future directions.

Advancements in AI for Pediatric Diagnostics and Prognostics

Many studies highlighted the possibility of AI in pediatric diagnostics. Using ML (Amrit et al., 2017) unstructured clinical data, it could be shown that ML models could accurately predict child abuse cases from unstructured clinical data [11]. Likewise, Ge et al. (2020) used the XGBoost model to predict PTSD in children and obtain diagnostic accuracies between 60 and 80% [12]. This shows that AI has the ability to derive useful insights from vast datasets and identify cases early enough for intervention [31].

One study focused on disease-specific applications, and there were other studies as well. Using an Adaptive Neuro-Fuzzy Inference System (ANFIS) model, Fathi et al. (2020) demonstrated the potential of AI for cancer diagnosis in pediatrics by predicting if a case is cancerous or not [13]. Zhu et al. (2020) showed that logistic regression models can screen for phenylketonuria (PKU), minimizing the false positive rate, implying that AI improves existing tools of diagnosis in their specificity and sensitivity [14].

AI also demonstrated power in predicting things over time through longitudinal studies. Pediatric refraction data from one decade ago were analyzed by Lin et al. (2018) to predict myopia progression over time in school-aged children here, showing that AI can forecast long-term disease development [15]. For instance, Radebe et al. (2021) employed random forest models to determine the predictability of thyroid nodule malignancy in children, which adds yet another level of AI’s capacity to support clinicians in high-risk case stratification [24]. All of these studies demonstrate that AI has lots of potential in pediatric diagnostics, but also safety concerns around model generalization, bias in the data set used to train the model, and the need for external validation. Because retrospective datasets depend on retrospective datasets, it can introduce potential biases to the AI that can be applied to different patient populations [3].

Ethical Considerations in AI-Driven Pediatric Medicine

The integration of AI in pediatric medicine poses vital ethical challenges, and this is based on data privacy, informed consent, and algorithmic bias [32]. As indicated by several literature reviews in this study, these dilemmas are quite ethical. The privacy of children’s data is very sensitive, so it has to be protected really strictly. Huang et al. and Hunt et al. warned of data breaches, unauthorized access, and misuse in just AI systems in pediatric healthcare [18,19]. From large datasets, many AI models are required to train, and thus, data anonymization, encryption, and state of affairs with regulations, including GDPR and HIPAA, are at the very top [33].

Shah et al. (2023) underscored the risks of cloud-based AI applications that enhance such threats from cyber exposure, especially when data is transferred from one institution to another [21]. Based on this, the study proposed that federated learning and blockchain technologies could address privacy risks by achieving decentralized data processing with confidentiality.

The challenge of informed consent is particularly acute with regard to pediatric AI applications. Parental consent is required for children as they cannot provide full informed consent. Anagnostopoulou et al. (2020) and Kalhori et al. (2021), however, also discussed the possibility that AI-made health decisions do not win the favor or even align with parental beliefs or preferences, thus breaching ethical boundaries [16,20]. If AI is being used to recommend early interventions for neurodevelopmental disorders, there’s a particular set of circumstances where this can lead to a conflict between medical paternalism and parental rights [34].

Xiao et al. (2018) further added that AI decisions that are far removed from conscious awareness, whether in terms of diagnoses, the diagnosis of other malfunctions, or recommendations on the diagnosis and treatment, make it impossible for guardians to fully appropriate AI-generated decisions [27]. However, explainable AI models are needed to address this “black box” problem to boost trust and enable people to make their decisions in an informed way. A particular area of concern within pediatric medicine is bias in AI models, as sociodemographic factors shape the patient’s health. Indeed, several studies mentioned that AI models trained on homogeneous datasets may not generalize to different pediatric populations, thus creating disparities in healthcare.

For instance, Shu et al. (2019) and McCartney (2018) raised concerns about racial and socioeconomic biases in AI-driven diagnostic tools, where underrepresented populations receive less accurate or delayed diagnoses [2,25]. AI models predominantly trained on Western datasets may not perform optimally in low and middle-income countries (LMICs), exacerbating global health inequities. Addressing these biases requires diverse, representative training datasets and continuous model validation across different populations [35].

Practical Challenges in AI Implementation in Pediatric Medicine

Despite AI’s potential, its integration into pediatric healthcare faces significant practical barriers related to infrastructure, clinician adoption, regulatory compliance, and cost. Implementing AI-driven healthcare solutions demands robust digital infrastructure, interoperability between electronic health records (EHRs), and high computational power. Clarke et al. (2022) and Vogl (2020) emphasized that many pediatric healthcare settings, especially in LMICs, lack the necessary technical infrastructure to support AI integration [22,26]. The high cost of AI model deployment further limits accessibility, restricting its benefits to well-funded healthcare systems [36].

Clinician skepticism toward AI remains a major barrier to widespread adoption. Several studies, including Aylward et al. (2022) and Davendralingam (2021), highlighted that lack of AI literacy, concerns about diagnostic accuracy, and fear of liability hinder acceptance among pediatricians [9,28]. Moreover, Hunt et al. (2020) and Filipow et al. (2022) noted that clinicians often struggle to interpret AI-generated recommendations, making it difficult to integrate AI insights into routine decision-making [19,23]. Training programs focusing on AI-assisted clinical workflows, explainable AI models, and real-world validation studies could enhance clinician confidence and adoption.

The evolving regulatory landscape poses another challenge for AI adoption in pediatric medicine. Most AI models used in research settings lack FDA or EMA approval for clinical deployment, raising concerns about legal accountability. Clarke et al. (2022) and Xiao (2018) stressed that regulatory bodies must establish clear guidelines for AI validation, risk assessment, and ethical governance to ensure safe implementation in pediatric care [22,27].

Limitations

Our systematic review has some limitations. Since only studies published in English were included in this systematic review, this may have excluded relevant studies that may have been published in other languages. This language restriction could introduce selection bias and limit the generalizability of the findings. A potential limitation of this review is the variability in methodological quality among the included studies, as identified through the Joanna Briggs Institute (JBI) Critical Appraisal Tools. While 17 studies were classified as low risk of bias, two exhibited moderate risk, and one had high risk, indicating potential concerns related to confounding factor management, statistical analysis, and study design. These variations may impact the overall strength of the evidence and should be considered when interpreting the findings.

Future Directions and Recommendations

Training the AI on a diverse, representative dataset will increase the generalizability of the model and ameliorate bias. Future work should be devoted to designing adaptive AI algorithms taking into account age-specific parameters and physiological variations. There are possibilities like federated learning, homomorphic encryption, and usage of blockchain-based healthcare records to reduce privacy risks and allow AI accessibility across healthcare institutions. Collaborative research with AI developers, pediatricians, bioethicists, and policymakers will help develop responsible AI roles. AI training modules can be incorporated into medical education to augment understanding of and incorporate AI tools into pediatric care. Robust Regulatory Frameworks: It is crucial that governments and health care agencies define strict legal and moral regulations for AI validation, liability, and child's rights.

## Conclusions

The studies analyzed in this systematic review illustrate various applications of AI in pediatric medicine, including disease diagnosis, predictive analytics, and clinical decision support. While individual studies demonstrate promising results, the overall impact remains contingent on further validation through large-scale, cumulative evidence. The responsible integration of AI requires a multidisciplinary approach that prioritizes transparency, patient-centered care, and regulatory oversight. Ensuring AI’s ethical and effective application in pediatric healthcare necessitates continuous advancements in algorithmic fairness, clinician training, and robust data security measures. Future research should focus on refining AI models for pediatric-specific conditions, fostering global collaboration, and addressing disparities in access to AI-driven healthcare. Ultimately, developing transparent, equitable, and clinically validated AI systems will be crucial for achieving improved patient outcomes while maintaining ethical integrity and public trust in AI-driven medical advancements.

## References

[1] Ullah W, Ali Q (2025). Role of artificial intelligence in healthcare settings: a systematic review. JMAI.

[2] Shu LQ, Sun YK, Tan LH, Shu Q, Chang AC (2019). Application of artificial intelligence in pediatrics: past, present and future. World J Pediatr.

[3] Indrio F, Pettoello-Mantovani M, Giardino I, Masciari E (2024). The role of artificial intelligence in pediatrics from treating illnesses to managing children's overall well-being. J Pediatr.

[4] Malhotra A, Molloy EJ, Bearer CF, Mulkey SB (2023). Emerging role of artificial intelligence, big data analysis and precision medicine in pediatrics. Pediatr Res.

[5] Otjen JP, Moore MM, Romberg EK, Perez FA, Iyer RS (2022). The current and future roles of artificial intelligence in pediatric radiology. Pediatr Radiol.

[6] Ramgopal S, Sanchez-Pinto LN, Horvat CM, Carroll MS, Luo Y, Florin TA (2023). Artificial intelligence-based clinical decision support in pediatrics. Pediatr Res.

[7] Lonsdale H, Jalali A, Ahumada L, Matava C (2020). Machine learning and artificial intelligence in pediatric research: current state, future prospects, and examples in perioperative and critical care. J Pediatr.

[8] Yu G, Li Z, Li S (2020). The role of artificial intelligence in identifying asthma in pediatric inpatient setting. Ann Transl Med.

[9] Aylward BS, Abbas H, Taraman S (2023). An introduction to artificial intelligence in developmental and behavioral pediatrics. J Dev Behav Pediatr.

[10] Page MJ, Moher D, Bossuyt PM (2021). PRISMA 2020 explanation and elaboration: updated guidance and exemplars for reporting systematic reviews. BMJ.

[11] Amrit C, Paauw T, Aly R (2017). Identifying child abuse through text mining and machine learning. Expert Sys Appl.

[12] Ge F, Li Y, Yuan M, Zhang J, Zhang W (2020). Identifying predictors of probable posttraumatic stress disorder in children and adolescents with earthquake exposure: a longitudinal study using a machine learning approach. J Affect Disord.

[13] Fathi E, Rezaee MJ, Tavakkoli-Moghaddam R, Alizadeh A, Montazer A (2020). Design of an integrated model for diagnosis and classification of pediatric acute leukemia using machine learning. Proc Inst Mech Eng H.

[14] Zhu Z, Gu J, Genchev GZ (2020). Improving the diagnosis of phenylketonuria by using a machine learning-based screening model of neonatal MRM data. Front Mol Biosci.

[15] Lin H, Long E, Ding X (2018). Prediction of myopia development among Chinese school-aged children using refraction data from electronic medical records: A retrospective, multicentre machine learning study. PLoS Med.

[16] Anagnostopoulou P, Alexandropoulou V, Lorentzou G (2020). Artificial intelligence in autism assessment. Int J Emerg Tech Learn (iJET).

[17] Brasil S, Pascoal C, Francisco R, Dos Reis Ferreira V, Videira PA, Valadão AG (2019). Artificial intelligence (AI) in rare diseases: is the future brighter?. Genes (Basel).

[18] Huang J, Shlobin NA, Lam SK, DeCuypere M (2022). Artificial intelligence applications in pediatric brain tumor imaging: a systematic review. World Neurosurg.

[19] Hunt X, Tomlinson M, Sikander S, Skeen S, Marlow M, du Toit S, Eisner M (2020). Artificial intelligence, big data, and mHealth: the frontiers of the prevention of violence against children. Front Artif Intell.

[20] Rostam Niakan Kalhori S, Tanhapour M, Gholamzadeh M (2021). Enhanced childhood diseases treatment using computational models: Systematic review of intelligent experiments heading to precision medicine. J Biomed Inform.

[21] Shah N, Arshad A, Mazer MB, Carroll CL, Shein SL, Remy KE (2023). The use of machine learning and artificial intelligence within pediatric critical care. Pediatr Res.

[22] Clarke SL, Parmesar K, Saleem MA, Ramanan AV (2022). Future of machine learning in paediatrics. Arch Dis Child.

[23] Filipow N, Main E, Sebire NJ, Booth J, Taylor AM, Davies G, Stanojevic S (2022). Implementation of prognostic machine learning algorithms in paediatric chronic respiratory conditions: a scoping review. BMJ Open Respir Res.

[24] Radebe L, van der Kaay DC, Wasserman JD, Goldenberg A (2021). Predicting malignancy in pediatric thyroid nodules: early experience with machine learning for clinical decision support. J Clin Endocrinol Metab.

[25] McCartney M (2018). Margaret McCartney: AI in medicine must be rigorously tested. BMJ.

[26] Vogl MT (2020). Artificial intelligence and organizational memory in government: the experience of record duplication in the child welfare sector in Canada. ACM DL.

[27] Xiao C, Choi E, Sun J (2018). Opportunities and challenges in developing deep learning models using electronic health records data: a systematic review. J Am Med Inform Assoc.

[28] Davendralingam N, Sebire NJ, Arthurs OJ, Shelmerdine SC (2021). Artificial intelligence in paediatric radiology: future opportunities. Br J Radiol.

[29] Ferrante G, Licari A, Fasola S, Marseglia GL, La Grutta S (2021). Artificial intelligence in the diagnosis of pediatric allergic diseases. Pediatr Allergy Immunol.

[30] Alsabri M, Aderinto N, Mourid MR (2024). Artificial intelligence for pediatric emergency medicine. J Med Surg Pub Health.

[31] Rasool S, Husnain A, Saeed A (2023). Harnessing predictive power: exploring the crucial role of machine learning in early disease detection. JURIHUM.

[32] Lu H, Alhaskawi A, Dong Y (2024). Patient autonomy in medical education: navigating ethical challenges in the age of artificial intelligence. Inquiry.

[33] Nankya M, Mugisa A, Usman Y (2024). Security and privacy in E-health systems: a review of AI and machine learning techniques. IEEE.

[34] Hadders-Algra M (2021). Early diagnostics and early intervention in neurodevelopmental disorders-age-dependent challenges and opportunities. J Clin Med.

[35] Krones F, Walker B (2024). From theoretical models to practical deployment: a perspective and case study of opportunities and challenges in AI-driven cardiac auscultation research for low-income settings. PLOS Digit Health.

[36] Udegbe FC, Ebulue OR, Ebulue CC (2024). The role of artificial intelligence in healthcare: a systematic review of applications and challenges. Int Med Sci Res Journ.

